# Integration of irradiation with cytoplasmic incompatibility to facilitate a lymphatic filariasis vector elimination approach

**DOI:** 10.1186/1756-3305-2-38

**Published:** 2009-08-14

**Authors:** Corey L Brelsfoard, William St Clair, Stephen L Dobson

**Affiliations:** 1 Department of Entomology, University of Kentucky, S-225 Ag. Science Center North, Lexington, KY 40546, USA; 2 Department of Radiation Medicine, Chandler Medical Center, 800 Rose Street, Lexington, KY 40536, USA

## Abstract

**Background:**

Mass drug administration (MDA) is the emphasis of an ongoing global lymphatic filariasis (LF) elimination program by the World Health Organization, in which the entire 'at risk' human population is treated annually with anti-filarial drugs. However, there is evidence that the MDA strategy may not be equally appropriate in all areas of LF transmission, leading to calls for the augmentation of MDA with anti-vector interventions. One potential augmentative intervention is the elimination of vectors via repeated inundative releases of male mosquitoes made cytoplasmically incompatible via an infection with *Wolbachia *bacteria. However, with a reduction in the vector population size, there is the risk that an accidental female release would permit the establishment of the incompatible *Wolbachia *infection type, resulting in population replacement instead of population elimination. To avoid the release of fertile females, we propose the exposure of release individuals to low doses of radiation to sterilize any accidentally released females, reducing the risk of population replacement.

**Results:**

*Aedes polynesiensis *pupae of differing ages were irradiated to determine a radiation dose that results in sterility but that does not affect the survival and competitiveness of males. Laboratory assays demonstrate that males irradiated at a female sterilizing dosage of 40 Gy are equally competitive with un-irradiated males. No effect of irradiation on the ability of *Wolbachia *to affect egg hatch was observed.

**Conclusion:**

An irradiation dose of 40 Gy is sufficient to cause female sterility, but has no observed negative effect on male fitness. The results support further development of this approach as a preventative measure against accidental population replacement.

## Background

Lymphatic filariasis (LF) is the leading cause of disability in South Pacific regions, where >96% of the 1.7 million population are at risk of LF infection [1]. The current World Health Organization (WHO) LF elimination campaign is premised upon the absence of a non-human reservoir for *Wuchereria bancrofti *and is focused on administering anti-filarial drugs to the 'at risk' human population via a mass drug administration (MDA) strategy [2-4]. However, MDA has not proven successful in eliminating LF from French Polynesia [2]. One hypothesis for the limited success of MDA is the complicated biology of the primary vector of *W. bancrofti *in French Polynesia: *Aedes polynesiensis*. Mosquitoes are required for the transmission of *W. bancrofti*. Unlike Culex and Anopheline vectors of LF, *A. polynesiensis *displays a pattern of negative density dependent transmission, allowing effective transmission of LF in low-level microfilaremics, which can result from MDA campaigns [5,6]. The stabilizing impact of negative density dependent transmission is hypothesized to be a contributing factor to an inability to eliminate LF in French Polynesia [2], leading to recommendations for the integration of vector control with MDA in areas where *A. polynesiensis *is the primary vector [2,5].

Control of *A. polynesiensis *has been attempted previously but has met with little success [7,8]. Vector control efforts are complicated by the inaccessibility of *A. polynesiensis *breeding sites and the geography of Pacific island nations [2,9]. While the geography of French Polynesia has complicated previous vector control efforts, it may facilitate an area wide elimination approach such as the sterile insect technique (SIT). Specifically, the population of vector mosquitoes is subdivided onto islands with limited immigration.

Multiple examples exist of successful SIT programs, including the New World screwworm, *Cochliomyia hominivorax *and the Mediterranean fruit fly, *Ceratitis capitata *[10]. Although mosquito SIT trials have been conducted, the results have been mixed, and the failures are often attributed to the relative fitness of released males [11]. SIT programs rely upon the delivery of sterile males that are competitive with wild type males for mates. Prior attempts based upon irradiation to sterilize males often resulted in the decreased fitness of release individuals [12-17]. While fitness decrease can be less of a problem with chemosterilization, concerns regarding non-target and environmental effects have led to their disuse [18]. For the above reasons, alternative forms of sterilization are receiving interest.

**Figure 1:**
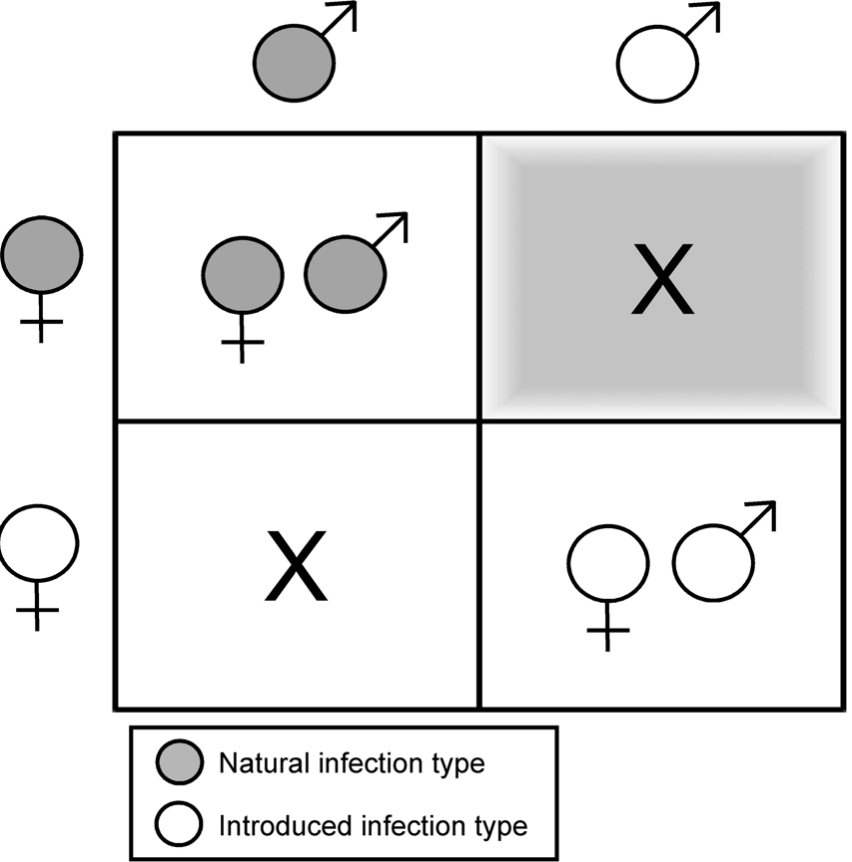
**Bi-directional cytoplasmic incompatibility**. Bi-directional CI results from incompatible crosses of individuals with different *Wolbachia *infection types indicated by an X, while individuals with the same *Wolbachia *type produce offspring with the same maternally inherited infection. The shaded box represents the applied IIT strategy where incompatible males with an introduced *Wolbachia *infection type are released into a population harboring a natural infection, resulting in sterility.

*Wolbachia pipientis *are intracellular maternally inherited bacteria that can cause multiple forms of host reproductive manipulation, including a form of sterility known as cytoplasmic incompatibility (CI). CI results in karyogamy failure and arrested embryonic development [19]. In populations where two incompatible *Wolbachia *types exist, bi-directional CI (bi-CI) can occur. With bi-CI, karyogamy failure and early embryonic arrest results in both cross directions between individuals with differing *Wolbachia *types (Figure 1). In a *Wolbachia *based vector control strategy, or incompatible insect technique (IIT), incompatibility is maintained by repeated inundative releases of incompatible males (Figure 1). Since *Wolbachia *is not paternally transmitted, the infection type present in the release strain does not become established in the field. As the size of the field population decreases, due to incompatible matings, an increasing proportion of males in the population are infected with the release *Wolbachia *type. Similar to conventional SIT, the increasing ratio of incompatible matings can lead to population elimination. The potential of the IIT strategy is demonstrated by a prior feasibility trial in Burma that was directed against a *Culex quinquefasciatus *population, in which the LF vector population was eliminated via repeated inundative releases of males infected with an incompatible strain of *Wolbachia *[20].

The success of an IIT strategy could be affected by the accidental release of females infected with the incompatible *Wolbachia *type. While the released females would be incompatible with wild type males, they would be compatible with released males. Thus, the accidental release of females could result in replacement of the targeted population with a population harboring the new *Wolbachia *infection type [21,22]. Recently, population replacement strategies have attracted much attention as a potential tool for modifying insect populations [22-24]. The prior trial in Burma relied upon sex separation techniques combined with visual sorting of release individuals to safeguard against female release [20]. While appropriate for a feasibility trial, manual inspection is unlikely to be cost effective at a large scale. To address this complication, we examine low-level irradiation as a means to sterilize any accidentally released female mosquitoes.

Previous studies have shown female mosquitoes are sterilized at a lower dose of irradiation than males. Importantly, at this sterilizing dose, male fitness (e.g., male mating competitiveness and/or longevity) is not adversely affected [16,17,25]. For the above reason, Curtis (1976) [26] suggested that release individuals (consisting primarily of cytoplasmically incompatible males) could receive a low dose of radiation to ensure sterility of any females that were not removed.

Here, we examine the strategy of pupal irradiation using a *Wolbachia *infected *A. polynesiensis *strain that is bi-directionally incompatible with naturally infected wild type mosquitoes [27]. We hypothesized that the incompatible *A. polynesiensis *strain could be irradiated as pupae at a dose sufficient to cause female sterility but that has little effect on survivorship, longevity, mating competitiveness of males, and the ability of *Wolbachia *to induce CI. Irradiation at the adult stage has been shown to result in fewer fitness consequences relative to individuals irradiated at the pupal stage [13,14,25], but irradiation of adults is often logistically difficult, since adults are fragile and cannot be easily manipulated in large quantities [28]. The results demonstrate that irradiated females have decreased fecundity and egg hatch with increasing radiation dose, and that female sterilization can be reached at a dose of 40 Gy. The latter dosage did not negatively impact survivorship, longevity, and mating competitiveness of males, nor did it affect the induction of CI. Results are discussed in relation to the integration of irradiation with the strategy of large-scale releases of cytoplasmically incompatible males to reduce the risk of unintentional population replacement.

## Methods

### Mosquito rearing

*A. polynesiensis *laboratory strains (AP) were obtained from French Polynesia and have been maintained in laboratory conditions for >50 generations. The bi-directionally incompatible *A. polynesiensis *strain (CP) is described in Brelsfoard et al. 2008 [27] and has been maintained in laboratory conditions for >20 generations. Mosquitoes used for experiments were reared in 21 cm × 21 cm × 7.5 cm disposable plastic pans (Pactive, Lake Forest, IL) at low densities (100-150 larvae per pan). Laboratory conditions consisted of a photoperiod of 18:6 Light:Dark at 29 ± 0.6°C, 73 ± 2.4% RH. Larvae were fed adlibitum 60 g/L liver powder suspension. One-week post emergence, females were allowed to blood feed on mice for 20 min (IACUC #00905A2005). Adults were provided a constant supply of 10% sucrose. Females were provided a 100 ml specimen cup lined with seed germination paper (Anchor Paper, St. Paul, MN) saturated with water for oviposition (this cup is referred to as the oviposition cup throughout the rest of the paper). Eggs were allowed to mature for seven days at 29 ± 0.6°C, 73 ± 2.4% RH. Following maturation, eggs were hatched in deoxygenated liver powder/water solution for 48 h.

### Irradiation procedure

Pupae were irradiated with electron beam radiation from a Varian 21EX linear accelerator (Varian Associates, Palo Alto, CA) at a rate of 4 Gy/min. Pupae were irradiated in 21 cm × 21 cm × 7.5 cm disposable plastic pans with a water level of 1 cm, placed 100 cm from the irradiation source, with 4 cm of Solid Water^® ^backscatter material (Gammex Inc., Middleton, WI) placed on top of the container. Female CP pupae originated from the same egg paper, but were hatched at different time points and were irradiated in two different age groups: early (16-24 hrs post pupation) and late (40-48 hrs post pupation), at three doses: 20, 30, and 40 Gy. For each dose and age group, 100-125 pupae were irradiated. CP males were irradiated as a group of 240 that were 30-48 hrs post pupation at a dose of 40 Gy. Both female age groups and males included a control treatment group (i.e., no irradiation), which were derived from the same pupae used for irradiation.

### Adult emergence rate

Following irradiation, pupae were placed individually in 13 × 100 mm culture tubes (Fisher Scientific, Pittsburgh, PA) to ensure virginity. Each tube received ~3 ml of de-ionized water. Tubes were checked at eight-hour intervals for adult emergence. Post emergence, the sex of each individual was confirmed and adults of the appropriate sex were transferred to treatment cages.

### Effect of irradiation on A. polynesiensis females

To examine CP female sterility and fecundity, 25 irradiated or un-irradiated CP females were transferred to cages with 25 un-irradiated CP males. Three days post blood feeding, females were provided an oviposition cup that was removed within ~48 hours. Individual eggs were scored as either hatching or non-hatching using a dissection scope (Leica MZ75, Bannockburn, IL). Four un-irradiated replicates and two replicates of each irradiation dose were maintained for each pupal age group. Per female fecundity was estimated by dividing the total number of eggs in the cage by the number of viable females at the time when oviposition cups were placed in cages. Sterility was estimated by determining the percent egg hatch of the egg paper removed from oviposition cups. To confirm insemination, spermathecae were checked in a subset of females by dissecting in Ringers solution and checking for the presence of sperm using an inverted compound microscope (Olympus IX70, Center Valley, PA). To examine survivorship, dead adults were removed from cages, their sex determined, and counted at 24-48 hour intervals.

### Effect of irradiation on A. polynesiensis males

In a subsequent experiment, 25 irradiated CP males were transferred to cages with 25 un-irradiated CP females. As a control, 25 un-irradiated CP males were transferred to cages with 25 un-irradiated CP females. Egg hatch, fecundity, and survivorship were determined using the same methods described for females.

To examine male mating competitiveness, ten virgin AP females were released into cages with 20 males of varying ratios of CP males [irradiated at 40 Gy or un-irradiated] and un-irradiated AP males. For both the irradiated and un-irradiated treatments, the AP:CP male ratios were: 20:0, 15:5, 10:10, 5:15, 0:20. Females were allowed to mate for five days and then blood fed. They were then isolated in oviposition cups and allowed to oviposit. Eggs were subsequently hatched, and the egg hatch rate was determined using a dissection scope. After hatching eggs, viable AP females producing non-hatching broods were dissected and their spermathecae examined for the presence of sperm. Females that were un-inseminated were excluded from the analyses.

### Statistical analysis

To analyze emergence rates, Wilcoxon/Kruskal-Wallis tests with a Bonferroni correction were performed to compare the number of adults emerging from irradiated treatments and un-irradiated controls. To analyze for differences in egg hatch, data were arcsin(sqrrt(x)) transformed and subjected to an ANOVA and followed by Tukey-Kramer tests with a Bonferroni correction to examine for differences between pairs of irradiation treatments. To analyze for differences in fecundity, data was subjected to an ANOVA and followed by Tukey-Kramer tests with a Bonferroni correction to examine for differences between pairs of irradiation treatments. Survival curves were generated and compared using the Kaplan-Meier method and log-rank tests to examine differences in adult longevity. To analyze male mating competitiveness, Chi-square goodness of fit tests were performed to compare the number of hatching broods for each ratio of irradiated and un-irradiated CP males to the expected number of hatching broods based on equal mating competitiveness. To examine for an effect of the ratio of irradiated and un-irradiated incompatible males on egg hatch, percent hatch data from all replicate cages were arcsin(sqrrt(x)) transformed and compared using a Wilcoxon/Kruskal-Wallis test. All statistical tests were performed using JMP 7.0.2 (SAS Institute, Cary, NC).

## Results

### Effect of irradiation on A. polynesiensis females

**Figure 2:**
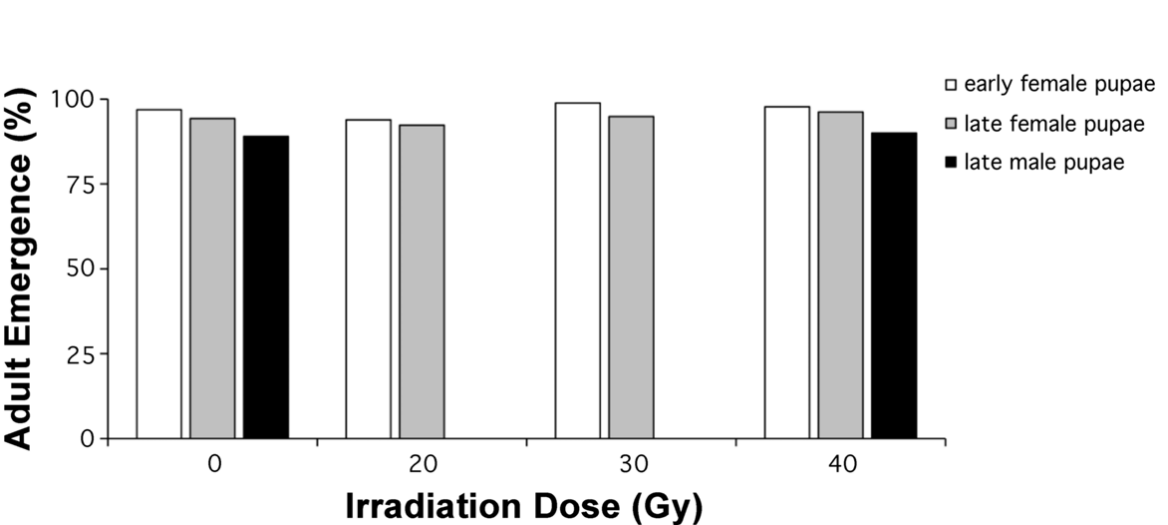
**The effect of a pupal irradiation dose on CP female and male adult emergence**.

As shown in Figure 2, irradiation of CP females had no effect on adult emergence when compared to controls (early, χ^2 ^= 2.68, df = 3, P > 0.44; late, χ^2 ^= 2.0, df = 3, P > 0.57). As shown in Table 1, egg hatch significantly decreased with increasing radiation dose for females irradiated at both pupal age classes (early, F = 60.3, df = 3,6, P < 0.0001; late, F = 39.0, df = 3,6, P < 0.0003). At 40 Gy, no eggs hatched for the early female pupae group. For the late female pupae group, egg hatch was reduced, with a mean hatch rate of 9.1% (Table 1). There was a trend for lower egg production with an increase in irradiation dosage, which was significant at 40 Gy for the early pupae group (early, F = 28.2, df = 3,6, P < 0.0007; late, F = 1.3, df = 3,6, P > 0.35) (Table 1). Only two eggs were produced in the 40 Gy treatment of early female pupae, and 59 eggs were produced in the 40 Gy treatment of late female pupae. For both female age classes, ≥75% of dissected females (n = 52) were positive for sperm in their spermathecae.

**Table 1:** The effect of irradiation on egg hatch and fecundity of *A. polynesiensis*.

Sex irradiated	Treatment dose (Gy)	*n*	Percent egg hatch ± SE	Egg number ± SE
**Early pupae**				
**Females**	0	4	77.9 ± 1.8^a^	53.1 ± 13.5^a^
	20	2	52.2 ± 15.0^ab^	17.6 ± 5^b^
	30	2	41.7 ± 1.0^b^	5.2 ± 2.9^b^
	40	2	0 ± 0^c^	0.06 ± 0.02^b^
**Late pupae**				
**Females**	0	4	82.6 ± 2.9^a^	44.7 ± 14.2^a^
	20	2	61.1 ± 6.0^ab^	32.9 ± 22.4^a^
	30	2	49.8 ± 10.2^b^	7.1 ± 3.9^a^
	40	2	9.1 ± 2.0^c^	1.4 ± 0.3^a^
**Late pupae**				
**Males**	0	3	64.7 ± 1.9^a^	28.2 ± 6.9^a^
	40	3	15.5 ± 2.6^b^	18.7 ± 4.5^a^

Within column mean values followed by different superscripted letters are statistically different (P < 0.017) using multiple comparison tests within each treatment group. *n *is the number of caged populations, which are the replicates of the experiment not the individuals in the cages.

Comparisons of females irradiated as early pupae demonstrated females irradiated at 20 Gy and 40 Gy had significantly decreased longevity compared to un-irradiated controls. At 30 Gy however, longevity was similar to controls (Table 2). Comparisons of female longevity demonstrated that females irradiated as late pupae at a 20 or 30 Gy irradiation dose did not have a significant impact on adult longevity (Table 2). However, a significant increase in longevity was observed when females in the late pupation group were irradiated at 40 Gy (Table 2).

**Table 2:** The effect of irradiation on mean survival times of *A. polynesiensis *males and females.

	Pupal age	Dose (Gy)	*n*	Mean ± SE survival time	Log rank
**Females**	Early	0	91	24.3 ± 1.3	n/a
		20	46	18.3 ± 1.9	6.96**
		30	31	24.0 ± 2.2	0.5
		40	46	17.7 ± 1.9	5.2*
					
	Late	0	81	22.2 ± 1.3	n/a
		20	38	25.3 ± 1.6	0.16
		30	45	24.0 ± 1.5	0.05
		40	45	29.0 ± 1.6	6.88**

**Males**	Late	0	72	7.9 ± 0.96	n/a
		40	73	10.7 ± 1.1	4.85*

*P < 0.05,**P < 0.01

### Effect of irradiation on CP males

After demonstrating female sterility at 40 Gy, a second experiment was conducted, examining males irradiated at the 40 Gy. As shown in Figure 2, irradiation of CP males had no effect on adult emergence when compared to controls (χ^2 ^= 1.0, df = 1, P > 0.32). Egg hatch decreased significantly when males irradiated at 40 Gy were mated with un-irradiated CP females (Table 1; F = 147.1, df = 1,4, P < 0.0004). A non-significant decrease in egg number was observed when CP females were mated with irradiated CP males (Table 1). Differences in adult longevity of males were also observed after irradiation. Male longevity increased significantly compared to un-irradiated controls when irradiated at 40 Gy (Table 2).

**Figure 3:**
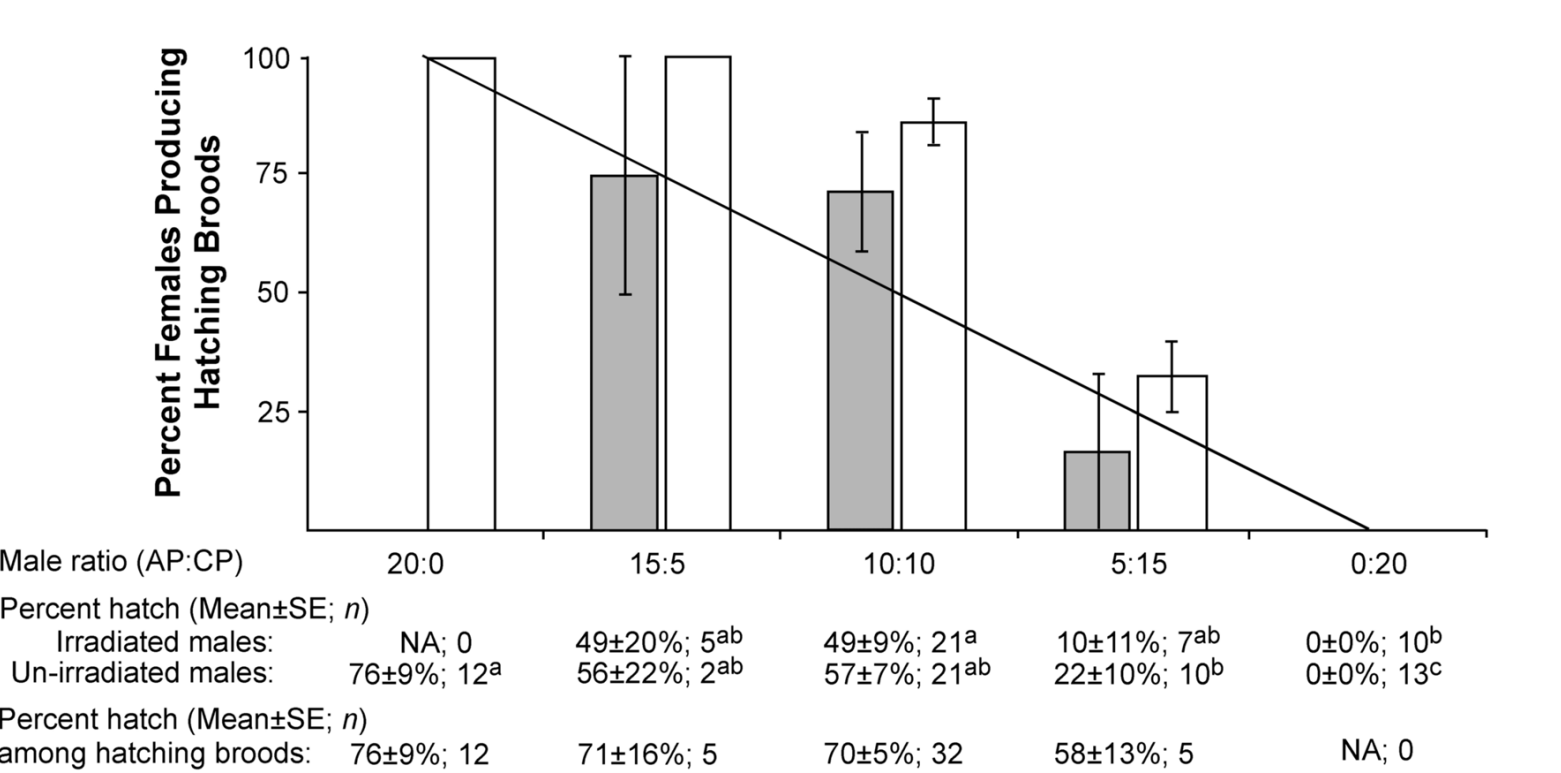
**The effect of irradiation dose on CP male mating competitiveness and *A. polynesiensis *population suppression**. Un-shaded and shaded bars represent cages with un-irradiated and irradiated CP males, respectively. Based upon the observed hatch rate, female broods were scored as either hatching (>6% hatch) or not hatching. The percent-hatching broods (hatching broods/total broods) were determined for each cage replicate. The solid line represents the expected values of compatibly mated broods assuming equal male mating competitiveness of the CP and AP males. Below the figure, egg hatch rates are based upon the combined oviposition of females in cages. Differing superscripted letters indicate significant differences within either the irradiated or un-irradiated treatments (Bonferroni corrected Wilcoxon/Kruskall Wallis tests; Irradiated CP males cages, P < 0.001; un-irradiated CP male cages, P < 0.002). The percent egg hatch among hatching broods was calculated to examine for intermediate hatch rates as an indicator of multiple insemination and fertilization of eggs by more than one male type.

No difference was observed in the numbers of observed and expected hatching broods for cages with irradiated (P > 0.25) and un-irradiated CP males (P > 0.05) relative to predictions based upon the assumption of equal male competitiveness of CP and AP males (Figure 3). Moreover, cages with only CP males and AP females demonstrated no loss of CI after a 40 Gy irradiation dose. Of 669 eggs examined from this cross, no hatching was observed.

Similar to female irradiation experiments, results suggest the majority of females in all treatments had mated. Insemination rates of virgin AP females were 90% for treatments with irradiated CP males (*n *= 21) and 89% for un-irradiated CP males (*n *= 21). Data also suggests females mated with only one male type in cages with a mixture of CP and AP males. There was no significant difference in the hatch rates of broods that were considered compatible (broods with a >6% hatch rate) for all treatments (χ^2 ^= 1.8, df = 3, P > 0.59) (Figure 3).

## Discussion

Adult emergence rates were used as a measure of survivorship after irradiation as pupae. A series of irradiation doses of up to 40 Gy had little effect on CP female adult emergence when they were irradiated as early and late pupae (Figure 2). Furthermore, a comparison of un-irradiated males with late CP male pupae irradiated at 40 Gy failed to detect an effect of irradiation on adult emergence rates (Figure 2). Results are consistent with previous studies that demonstrate high (>90%) adult emergence rates when mosquito pupae were irradiated at 16 hrs post pupation or older [12,14]. Overall, data suggests irradiation has no effect on adult emergence.

An examination of egg hatch from different irradiation doses demonstrates that CP females become sterile at a dose of 40 Gy, when irradiated as early pupae. However, low levels of egg hatch from CP females were observed when irradiated at 40 Gy as late pupae. Fecundity decreased with irradiation dose for both age classes of female pupae. However, CP females were observed to be more fecund when irradiated as late pupae. A decrease in egg number due to irradiation has been shown to be associated with cellular changes in germ line cells, more specifically developing follicle cells of the ovaries [25]. Results observed here are similar to a previous study where *Aedes aegypti *egg production was entirely eliminated after exposure to a sterilizing dose of irradiation [29]. Furthermore, insemination rates of females were high (>75%), suggesting the loss of fecundity and fertility of CP females is the result of irradiation treatments not a loss of insemination by CP males. The latter 'lower impact to late pupae' is consistent with prior studies. Specifically, somatic and germline cells undergoing meiosis and mitosis are the most sensitive to irradiation. Older pupae have fewer somatic mitotic cells and a more developed germline, resulting in lessened effects of irradiation [25,28]. Here it is hypothesized that germline damage played a more significant role in the observed decreases in egg hatch and fecundity than somatic damage.

The effects of irradiation on germ line cells and somatic cells can also cause damage that results in decreased longevity [30]. Prior studies with mosquitoes have shown a decrease in adult longevity after irradiation of *A. aegypti *pupae at a dose of 40 Gy and higher [25] and for *Anopheles pharoensis *and *Anopheles stephensi *irradiated at >80 Gy [12,15]. While a general trend toward increased mortality among irradiated individuals was observed, there are notable exceptions. Specifically, significantly increased longevity was observed in some irradiated treatments relative to the control individuals. While initially counterintuitive, similar results have been reported for *Anopheles arabiensis *irradiated at 35-80 Gy [14] and *A. pharoensis *irradiated at 5-70 Gy [31]. The observed increase in male longevity resulting from irradiation merits additional study. However, relevant to the applied strategy discussed here, there was no observed decrease in male longevity. Thus, a low level of irradiation is not observed to reduce male longevity, which could decrease the effectiveness of the proposed strategy.

In a subsequent experiment examining the effects of irradiation on male mating competitiveness, irradiated and un-irradiated CP males were allowed to compete for mates against AP males. The results demonstrate that CP males do not differ from AP males in their competitiveness, regardless of whether irradiated or un-irradiated (Figure 3). Male mating competitiveness results are similar to those observed in previous studies examining the male mating competitiveness of CP males with AP males [27]. Furthermore, a difference was not noted in a comparison of spermathecae from AP females mated with either irradiated or un-irradiated CP males, demonstrating that male irradiation did not affect insemination rates. Furthermore, no eggs hatched in crosses of irradiated CP males with AP females, suggesting irradiation did not affect the ability of *Wolbachia *to induce CI. If there were an effect of irradiation on the CI mechanism, hatching eggs would be expected from this cross.

The experimental design permits the investigation of a population elimination strategy, based upon incompatible male releases. As shown in Figure 3, the percent egg hatch decreased from 76 to 0% for both the irradiated and un-irradiated CP males, inversely related to the ratio of incompatible males. It is emphasized that the experiments described here were performed in small laboratory cages under optimal conditions. Field cage tests are required to better understand the survivorship and mating competitiveness of irradiated males.

An additional concern of this strategy is AP females using sperm from more than one male type to fertilize their eggs. A comparison of egg hatch rates resulting from compatibly mated females did not differ significantly between treatments. If females were utilizing sperm to fertilize their eggs from more than one male type, intermediate hatch rates would be expected in cages with a mixture of CP and AP males. The results are consistent with previous studies, suggesting *A. polynesiensis *females utilize sperm from one male type [27].

While the removal of females from release material continues to be an important component, the strategy described here can reduce the probability of accidental population replacement. However, unintentionally released females will bite and have the potential to transmit disease. On the other hand, the observed decrease in adult female longevity after irradiation could reduce this risk, since shorter-lived females are less likely to complete the required extrinsic incubation period.

Since the goal of the strategy is to maximize impact on females, irradiation of females <24 hrs post-pupation is optimal. Assuming appropriate rearing practices, cohorts of immatures should be relatively synchronized. Since males pupate before females [32], collections can be timed such that they consist primarily of late male pupae and early female pupae. Males and females can be quickly and easily separated based upon size using a mechanical sorting device. The resulting pool would primarily consist of late male pupae and a few early female pupae. The results shown here suggest that this would be ideal for minimizing effects on males, while maximizing female sterility.

## Conclusion

The work presented here supports the integration of irradiation with CI as part of an IIT strategy targeting LF transmission by *A. polynesiensis *in French Polynesia. With the integration of low doses of irradiation, accidentally released females would be sterile, reducing the risk of unintentional population replacement. While low dose irradiation only partially sterilizes males, the released males are fully incompatible with wild type females due to *Wolbachia*-induced bi-CI. While the complete removal of females from release material remains the goal, the described approach could obviate the requirement of visually checking release material to remove all females. The latter manual checks are expected to be the most cost prohibitive barrier to large-scale implementation of the strategy.

It is realized that an alternate irradiation device is needed to facilitate the irradiation of large numbers of pupae. Furthermore, methods of irradiation will also have to conform to regulations in French Polynesia. X-ray irradiators are available from multiple companies, which will likely provide a more acceptable and feasible option than radioactive isotopes. Future studies should examine improved devices for irradiating *A. polynesiensis *(e.g., different energy sources). Studies should also be performed at a larger scale, with multiple replications of the 40 Gy irradiation dose. In addition, experiments should ultimately be performed in semi-field conditions that compare the mating competitiveness and survivorship of irradiated CP males with wild type *A. polynesiensis *collected from the field.

## Abbreviations

LF: Lymphatic filariasis; MDA: Mass drug administration; CI: Cytoplasmic incompatibility; IIT: incompatible insect technique; SIT: sterile insect technique; bi-CI: bi-directional CI; CP: hybridized *A. polynesiensis *(Described in Brelsfoard et al. 2008) [27]); AP: wild type *A. polynesiensis*; Gy: Gray; a SI unit of absorbed radiation.

## Competing interests

The authors declare that they have no competing interests.

## Authors' contributions

CLB and SLD conceived and designed the experiments. CLB and WSC performed experiments. CLB analyzed the data. CLB and SLD wrote the paper.
